# Genetic Diversity of Newcastle Disease Virus in Brazilian Pigeons and Expanded Phylogenetic Characterization of Sub-Genotype VI.2.1.2

**DOI:** 10.3390/v18101130

**Published:** 2026-10-12

**Authors:** Laura Morais Nascimento Silva, Andreina de Carvalho Araujo, Talita Dayane Pereira e Silva, Andrew Y. Cho, Juliana A. Sinhorini, Luiz Alfredo Martins Lopes Baptista, Terezinha Knöbl, Guilherme Pereira Scagion, Bruna Larotonda Telezynski Lopes, Larissa Mayumi Bueno, Luciano Matsumiya Thomazelli, Alessandra Greatti, Meriane Demoliner, Micheli Fillipi, Vyctoria Malayhka de Abreu Góes Pereira, Jansen de Araújo, Sun-Hak Lee, Clarice Weis Arns, Edison Luiz Durigon, Maria Clorinda Soares Fioravante, Fernando Rosado Spilki, Dong-Hun Lee, Kiril M. Dimitrov, Helena Lage Ferreira

**Affiliations:** 1 Graduate Program in Experimental Epidemiology and One Health, School of Veterinary Medicine and Animal Science, University of São Paulo, Av. Prof. Orlando Marques de Paiva, 87, Butantã, São Paulo 05508270, SP, Brazil; lauramonasc@usp.br; 2 Department of Veterinary Medicine, Faculty of Animal Science and Food Engineering, University of São Paulo, FZEA-USP, Duque de Caxias Norte Avenue, Pirassununga 13635900, SP, Brazil; andreinacarvalhoa@usp.br (A.d.C.A.); gui.scagion@gmail.com (G.P.S.); buenolm@usp.br (L.M.B.); 3 Graduate Program in Animal Science, Veterinary and Animal Science School, Federal University of Goias, EVZ/UFG, Rodovia Goiânia/Nova Veneza (GO 462), km 0, Goiânia 74001970, GO, Brazil; talita.vet@hotmail.com (T.D.P.e.S.); clorinda@vet.ufg.br (M.C.S.F.); 4 College of Veterinary Medicine, Konkuk University, 120 Neungdong-ro, Gwangjin-gu, Seoul 05029, Republic of Korea; 7rewcho@gmail.com (A.Y.C.); cnescis@naver.com (S.-H.L.); donghunlee@konkuk.ac.kr (D.-H.L.); 5 Zoonoses Surveillance Division of the Health Surveillance Coordination, Health Department of São Paulo, São Paulo 02039020, SP, Brazil; janaya@prefeitura.sp.gov.br; 6 Wild Animal Screening Center (CETAS-GO), Rua José Alves Toledo, Chácara da União, Goiânia 74684021, GO, Brazil; luiz.baptista@ibama.gov.br; 7 Avian Medicine Laboratory, Veterinary Medicine and Animal Science School, University of São Paulo, Av. Prof. Orlando Marques de Paiva, 87, Butantã, São Paulo 05508270, SP, Brazil; tknobl@usp.br; 8 Institute of Biomedical Sciences, University of São Paulo, Av. Prof. Lineu Prestes 1374, São Paulo 05508-000, SP, Brazil; bruna.larotonda.lopes@usp.br (B.L.T.L.); lucmt@usp.br (L.M.T.); jansentequila@usp.br (J.d.A.); eldurigo@usp.br (E.L.D.); 9 Department of Genetics, Evolution and Bioagents, Institute of Biology, University of Campinas—UNICAMP, Campinas 13083-970, SP, Brazil; alessandragreatti@yahoo.com.br (A.G.); clarns@gmail.com (C.W.A.); 10 Institute of Health Sciences, Feevale University, Novo Hamburgo 93525075, RS, Brazil; merianedemoliner@gmail.com (M.D.); michelifilippi@hotmail.com (M.F.); vyctoriamalayhkaa@gmail.com (V.M.d.A.G.P.); fernandors@feevale.br (F.R.S.); 11 Texas A&M Veterinary Medical Diagnostic Laboratory, TVMDL, College Station, TX 77843, USA; kiril.dimitrov@tvmdl.tamu.edu

**Keywords:** columbiformes, emerging, Newcastle disease, viruses, molecular surveillance, phylogenetic analysis

## Abstract

Pigeon paramyxovirus 1 (PPMV-1) comprises pigeon-associated variants of Newcastle disease virus (NDV), predominantly belonging to genotypes VI, XX, and XXI. These viruses have been detected in feral pigeons in Brazil since at least 2014, and an NDV outbreak associated with a pigeon-origin genotype VI virus was reported in commercial broilers in 2024. Here, we characterized two PPMV-1 strains detected in pigeons samples from geographically distinct regions of Brazil. Virus BR/105/21 was detected in Midwestern Brazil, whereas BR/34/22 was detected in Southeastern Brazil. Genome sequencing yielded a complete genome sequence for BR/105/21 (15,192 nt; 100% reference-genome coverage; mean depth, 3334×) and a near-complete genome sequence for BR/34/22 (15,133 nt; 99.5% reference-genome coverage; mean depth, 20.8×). Both viruses possessed the F-protein cleavage-site motif ^113RQKR↓F^117, which is consistent with the molecular criterion for virulent NDV. Phylogenetic analysis based on the complete F-gene coding sequence grouped both viruses with PPMV-1 previously detected in Brazil between 2014 and 2019. Analysis of VI.2.1.2 sequences identified three well-supported phylogenetic groups with between-group nucleotide distances of 6.1–8.0%. Based on established NDV classification criteria, two groups are proposed as VI.2.1.2.1, comprising viruses from Nigeria detected between 2007 and 2015, and VI.2.1.2.2, comprising Brazilian viruses detected between 2014 and 2022 and one virus detected in Argentina in 1997. These findings expand the available genetic data for pigeon-associated NDV in South America and reveal substantial genetic heterogeneity and phylogenetic structure within VI.2.1.2, reinforcing the importance of genomic surveillance of NDV in pigeons.

## 1. Introduction

Orthoavulavirus javaense, commonly known as Avian paramyxovirus 1 (APMV-1) or Newcastle disease virus (NDV, used hereafter), belongs to the genus Orthoavulavirus of the family Paramyxoviridae. NDV is classified into two distinct genetic classes, I and II [1], within a single serotype. Class II viruses have high genetic diversity and are further divided into at least 20 genotypes [2]. Viruses in domestic and wild birds can infect over 250 bird species with variable pathogenicity. In poultry, virulent NDV is responsible for devastating outbreaks [3]. The World Organisation for Animal Health (WOAH) defines virulent NDV isolates as those with intracerebral pathogenicity index (ICPI) values over 0.7 and/or a fusion (F) protein cleavage site containing a polybasic amino acid motif [4].

Pigeon-associated variants of NDV are commonly referred to as pigeon paramyxovirus 1 (PPMV-1). PPMV-1 is diverse and is classified into genotypes VI, XX, and XXI [2]. PPMV-1 was historically recognized as an antigenic variant of NDV based on a characteristic monoclonal-antibody reactivity pattern [5]. However, the current genetic classification of pigeon-associated NDV encompasses viruses belonging predominantly to genotypes VI, XX, and XXI, and not all viruses assigned to these genotypes have been characterized using the historical MAb panel [6]. Thus, the designation PPMV-1 is commonly used for pigeon-associated NDV within these genetic groups rather than being defined solely by monoclonal-antibody reactivity. PPMV-1 is maintained and is endemic in feral and wild columbids and causes neurological disorders with high mortality rates when infecting these hosts [7]. First emerging in the Middle East in the late 1970s, PPMV-1 has been found worldwide [7,8]. Although PPMV-1 commonly possesses a polybasic F-protein cleavage site consistent with the WOAH molecular criterion for virulent NDV, pigeon-origin viruses may show lower pathogenicity and transmission efficiency in chickens than chicken-adapted virulent NDV [9], and variable ICPI values have been reported [10]. Experimental passage in chickens can select viral variants with increased replication and pathogenicity [11].

Brazil is the second-largest poultry meat producer and the largest exporter of poultry meat worldwide [12]. NDV vaccination with live lentogenic viruses of genotypes I and II is compulsory for commercial breeders and layers and optional for broilers. An NDV outbreak caused by genotype V viruses was reported in 2006 in backyard flocks. In 2024, Brazil reported an outbreak in 14,400 susceptible commercial broilers. According to the official report [13], the outbreak was caused by a virus that shared greater than 95% fusion gene nucleotide identity with a PPMV-1 previously identified in Southern Brazil in 2014 [14]. Nevertheless, the genome sequence of the virus causing the 2024 outbreak has not been made available yet.

Despite recurrent reports of mortality in feral pigeons in different Brazilian regions, genomic information on pigeon-associated NDV in Brazil remains limited, with only a small number of sequences reported since 2014 [14,15,16]. This limited genetic information constrains the characterization of the diversity and geographic distribution of these viruses and is particularly relevant following the detection of genotype VI NDV in commercial poultry in 2024. In the present study, we characterized two pigeon-origin NDV strains detected in southeastern and central-western Brazil in 2021 and 2022, respectively. We obtained their genome sequences and analyzed their phylogenetic relationships with pigeon-associated NDV from Brazil and other countries, with particular emphasis on the genetic structure and classification of sub-genotype VI.2.1.2.

## 2. Materials and Methods

### 2.1. Samples

Samples from two pigeons were investigated in this study. The samples analyzed in this study originated from two independent diagnostic/surveillance events and were not collected as part of a randomized epidemiological survey. The first sample was a lung obtained from a picazuro pigeon (Patagioenas picazuro), labeled BR/105/21, that died in March 2021 after being rescued at the Centro de Triagem de Animais Silvestres de Goiânia (CETAS—GO), a sorting center for wild animals located in Midwestern Brazil (−16.59845, −49.19883). For the second case, different tissues, including kidney, proventriculus, brain, heart, intestine, lung, and trachea, were collected during necropsy from a pigeon (Columba livia), labeled BR/34/22, that died in May 2022 during an outbreak in pigeons with neurological clinical signs compatible with NDV infection in the Zoonose Surveillance Division (DVZ) of the Health Surveillance Coordination, São Paulo Municipal Health Department, Southeastern Brazil. This is the same location (−23.5121998, −46.6301312) as a previous report from 2019 [16]. Sample collection, virus characterization, and virus isolation were approved by and performed following the Institutional Animal Care and Use Committee (IACUC) (CEUA-FZEA-USP: CEUA1355190521 and CEUA-UFG: MB 021/2020).

### 2.2. RNA Extraction and Real-Time RT-PCR (rRT-PCR)

Molecular detection of NDV RNA in the original tissue samples was performed by rRT-PCR prior to virus isolation. Briefly, RNA was isolated from the collected tissues using the MagMax Core™ Total Nucleic Acid Isolation Kit (Thermo Fischer Scientific, Carlsbad, CA, USA), according to the manufacturer’s instructions. Real-time RT-PCR targeting the NDV matrix (M) and F genes [17] was performed using the AgPath-ID One-Step RT-PCR (Thermo Fisher Scientific, MA, USA) kit in a volume of 25 μL containing 12.5 μL of master mix, 0.25 μL of RT enzyme, 0.125 μL of each oligo (20 pmol/uL of each oligonucleotide), 0.025 μL of probe (6pmol/uL of probe) and 5 μL of RNA. Reactions were performed using one cycle of 30 min at 50 °C and 15 min at 95 °C, followed by 45 cycles of 1 s at 94 °C and 1 min at 60 °C, using a QuantStudio 5 thermocycler (Applied Biosystems, Darmstadt, Germany).

### 2.3. Viral Isolation

Viral isolation was performed in 9- to 11-day-old embryonated chicken eggs (negative for APMV-1) following the standard methodology described in the WOAH manual [10]. Inocula comprised homogenized lung tissue from bird BR/105/21 and homogenized pooled kidney and proventriculus tissues from bird BR/34/22.

### 2.4. Complete Genome Sequencing and Assembly

Genome amplification was conducted on total RNA purified from allantoic fluids using the SuperScript IV Reverse Transcriptase kit (Thermo Fisher Scientific, USA) with random hexamers [16]. Library preparation was done using the Nextera XT DNA kit (Illumina). Next-generation sequencing was performed on the NextSeq 550 instrument (Illumina, San Diego, CA, USA) using the NextSeq 500/550 Mid Output Kit v2 (300 cycles). Genome assembly was done using a “map-to-reference” approach. Reads were mapped to the NDV reference genome MZ458602 using the Geneious (2026.0.2) mapper implemented in Geneious Prime, with the Medium Sensitivity/Fast setting and fine-tuning for up to five iterations. Reference-guided assemblies were inspected for genome coverage and read depth. The BR/34/22 consensus sequence and read mapping were further examined in Geneious Prime to identify ambiguous nucleotide positions and assess sequencing depth across the complete F-gene coding sequence used for phylogenetic inference. Independent sequencing replication was not performed. Assembled genomes were deposited in GenBank under the accession numbers PQ212861 and PQ212862.

### 2.5. Phylogenetic Analysis

Recombination screening was performed on the complete F-gene alignment using the Recombination Detection Program (RDP) v4.101. Seven methods implemented in RDP4 (RDP, GENECONV, BootScan, MaxChi, Chimaera, SiScan, and 3Seq) were used to screen for potential recombination signals. Sequences were treated as linear, and statistical significance was assessed at *p* < 0.05 with Bonferroni correction for multiple comparisons. Candidate signals were subsequently evaluated based on concordance among detection methods and inspection of the associated phylogenetic patterns. The estimates of average evolutionary distances between different sub/genotypes of NDV and the isolates in the present study were calculated with MEGA6 [18] using the Maximum Composite Likelihood model [19]. The rate variation among sites was modelled with a gamma distribution (shape parameter = 1). For comprehensive phylogenetic analysis, the dataset of complete F-gene sequences provided by the international consortium that published the current NDV classification system [2] was used (dataset deposited in GitHub Desktop 3.6.7-beta3 at https://github.com/NDVconsortium/NDV_Sequence_Datasets, accessed on 13 January 2026). The sequences of the two isolates studied here were added to the dataset. A BLAST+ 2.17.0 search showed that the most closely related sequences available in GenBank are already part of the dataset. All sequences (n = 2159) were aligned using Multiple Alignment with Fast Fourier Transformation (MAFFT v7.4.50) [20]. Next, a preliminary maximum-likelihood tree was built using RaxML version 8.2.11 [21] based on the general time-reversible (GTR) model [22] (goodness of fit was measured by the corrected Akaike information criterion in MEGA 6). Based on the topology and distance analyses of the studied isolates, a maximum-likelihood tree with 1000 bootstrap replicates was built using RaxML on a subset of a total of 460 sequences of genotypes VI, XX, and XXI and four outgroup sequences. Evolutionary rate differences among sites were modelled with a discrete Gamma distribution (Γ), and the rate variation model allowed for some sites to be evolutionarily invariable (I). The RaxML tree was constructed through the Geneious Prime software. The tree was visualized using FigTree v1.4.4 (http://tree.bio.ed.ac.uk/software/figtree, accessed on 13 January 2026). The taxa names include a Roman–Arabic numeral representing the respective genotype for each isolate, the GenBank accession number, host name (if available), country of isolation, isolate designation, and year of isolation. The classification and nomenclature criteria put forth by the NDV consortium [2] based on phylogenetic topology and evolutionary distances between different taxonomic groups were used for sub/genotype identification.

## 3. Results

### 3.1. Molecular Detection

The rRT-PCR performed on the lung sample from bird BR/105/2021 yielded positive results for both M and F genes, with Ct values of 25.2 and 37.0, respectively. For bird BR/34/22, macroscopic lesions included lung congestion, diffuse hepatitis, mild necrosis, and hemorrhages in the pancreas, duodenum, and brain. The samples from BR/34/22 were positive by rRT-PCR for the M gene in all tissues, with Ct values of 27.9, 28.4, 28.7, and 29.4 for the lung/trachea, heart, intestine, and brain, respectively, indicating a generalized distribution of the virus. The F-gene rRT-PCR targeting a virulent-NDV-associated sequence was also positive, although with high Ct values (>37) in the lung, trachea, heart, and intestine samples. As the fusion gene of PPMV-1 shows genetic differences compared to the chicken-origin F genes (the F-gene rRT-PCR test is designed to detect the latter), it is not surprising that the F-gene Ct values were higher than the values for the matrix-gene assay. These differences may affect assay sensitivity, as previous studies have reported false-negative results for some PPMV-1 strains [23]. Inoculation of embryonated chicken eggs resulted in the isolation of hemagglutinating viruses.

### 3.2. Genome Analyses

Whole-genome sequencing yielded a near-complete genome sequence for BR/34/22 (15,133 nt) and a complete genome sequence for BR/105/21 (15,192 nt). Reference-guided assembly yielded 2523 mapped reads for BR/34/22, with a mean depth of 20.8× and 99.5% reference-genome coverage (15,119/15,192 nt), whereas BR/105/21 yielded 328,278 mapped reads, with a mean depth of 3334× and complete coverage of the 15,192-nt reference genome. The consensus sequences deposited in GenBank were used for subsequent genetic analyses. Both genomes showed the typical NDV genome organization, 3′-NP-P-M-F-HN-L-5′. The polybasic amino-acid motif ^113RQKR↓F^117 was identified at the F-protein cleavage site of both viruses, consistent with the WOAH molecular criterion for virulent NDV [10]. Further examination of the BR/34/22 consensus sequence identified a single ambiguous nucleotide (R, A/G) at genome position 4475, located in the intergenic region between the M and F genes. The F-gene coding sequence used for phylogenetic analysis contained no ambiguous nucleotide positions and showed a mean sequencing depth of 24.4× (range: 8–50×). Thus, the identified ambiguity was located outside the region used for phylogenetic inference and genetic-distance calculations.

### 3.3. Phylogenetic Analyses

**Figure 1:**
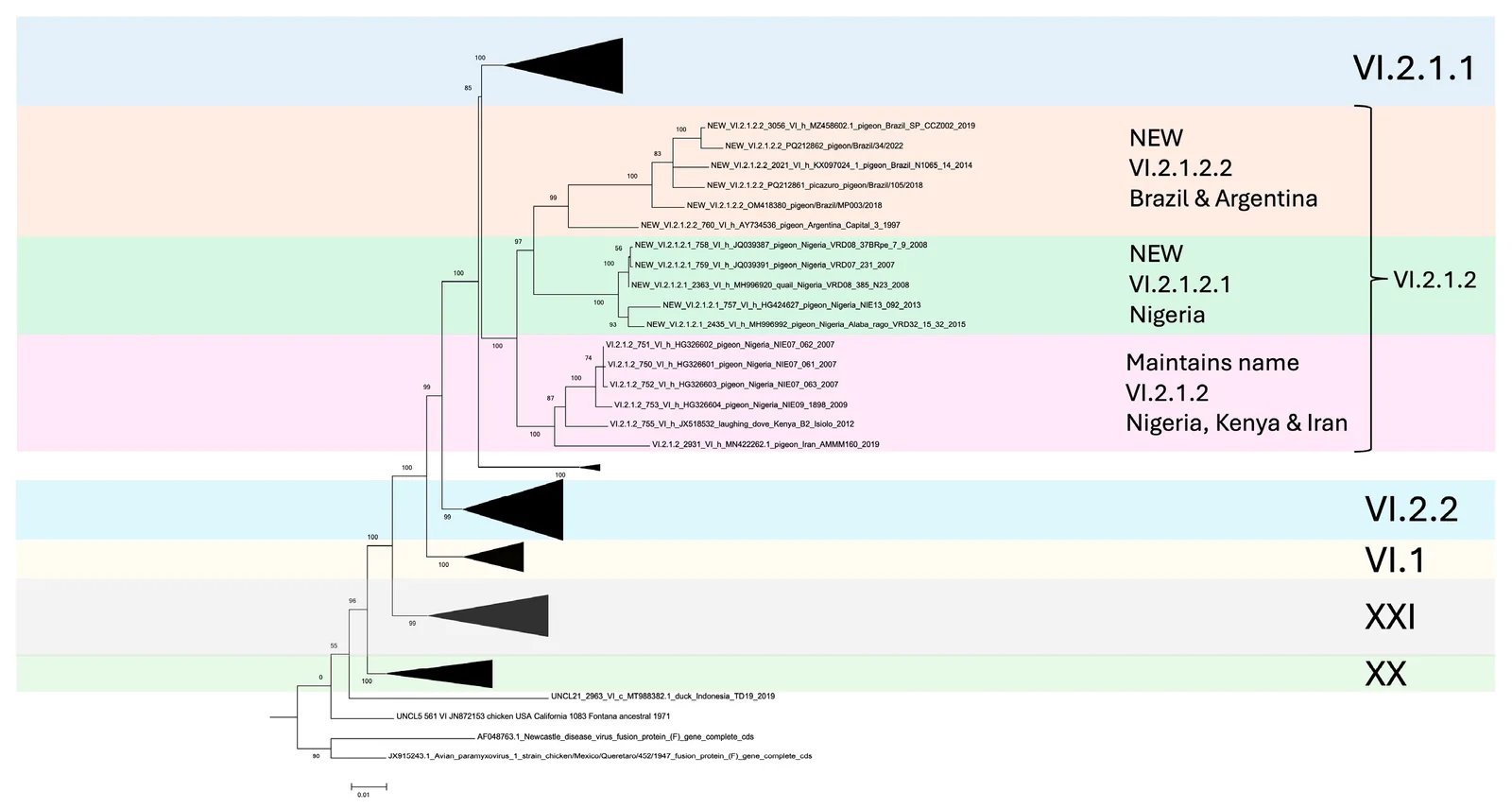
Phylogenetic tree based on the complete fusion (F) gene of Newcastle disease virus (NDV) class II, including genotypes VI, XX, and XXI (n = 464; collapsed view). The analysis identified two newly proposed sub-genotypes within VI.2.1.2: VI.2.1.2.2 (highlighted in orange), which includes the two isolates characterized in this study, clustered with three Brazilian viruses (2014–2019) and one pigeon isolate from Argentina (1997); and VI.2.1.2.1 (highlighted in green), comprising Nigerian isolates collected between 2007 and 2015. The remaining VI.2.1.2 sequences (highlighted in pink) include viruses from Nigeria, Kenya, and Iran (2007–2019). Bootstrap values are indicated at major nodes. The tree was constructed using the maximum-likelihood method, with branch lengths proportional to the number of nucleotide substitutions per site.

Recombination screening did not identify supported recombination events involving either BR/105/21 (PQ212861) or BR/34/22 (PQ212862). Candidate recombination signals were detected among other sequences in the dataset; however, these signals showed limited or inconsistent support across detection methods and were therefore not considered robust evidence of recombination. Phylogenetic analysis based on the complete F gene grouped the two isolates studied here with three sub-genotype VI.2.1.2 viruses isolated in Brazil between 2014 and 2019 (Figure 1). These five Brazilian sequences formed a monophyletic branch with a single sequence from a pigeon isolated in Argentina in 1997 (temporarily named here as Group 1). The remaining sub-genotype VI.2.1.2 sequences formed two separate monophyletic branches with high node support (>97). One comprised five sequences from viruses isolated in Nigeria between 2007 and 2015 (temporarily named here as Group 2). The other branch consisted of six sequences, four of which were from Nigerian viruses (2007–2009), one from Kenya in 2012, and one from Iran in 2019 (temporarily named Group 3) (Figure 1). The pairwise distance analyses show that the Brazilian sequences shared 96.7% to 99.2% nucleotide identity (Appendix A). The three groups formed distinct monophyletic branches with bootstrap support of 99%, 97%, and 100%, respectively. The three groups of sequences described above showed high genetic divergence, with between-group nucleotide distances varying from 6.1% to 8.0%. Their nucleotide distances to all other genotype VI sub-genotypes varied between 6.1% and 10% (Table 1). Each group comprised at least four epidemiologically independent isolates from distinct outbreaks. These findings fulfilled the established criteria for sub-genotype differentiation proposed by Dimitrov et al. (2019) [2], supporting the proposed designations VI.2.1.2.1 (Group 2) and VI.2.1.2.2 (Group 1), while Group 3 retains the designation VI.2.1.2.

## 4. Discussion

Phylogenetic analyses revealed substantial genetic diversity within the previously recognized sub-genotype VI.2.1.2, supporting its subdivision into three distinct groups based on the classification criteria established by Dimitrov et al. (2019) [2]. Based on their phylogenetic relationships, genetic distances, and epidemiological independence, two additional sub-genotypes are proposed: VI.2.1.2.1, comprising viruses from Nigeria, and VI.2.1.2.2, comprising viruses from Brazil and Argentina. The third group, including viruses from Nigeria, Kenya, and Iran, retains the original VI.2.1.2 designation. Notably, the Argentine isolate showed nucleotide distances of 6.0–6.7% from the Brazilian viruses, indicating substantial genetic diversity within the proposed VI.2.1.2.2 group. The Brazilian viruses were detected in geographically separated regions and different years, documenting the presence of genetically related pigeon-associated NDV in multiple locations across Brazil. However, the limited number of available sequences and the reliance on independent diagnostic and surveillance events, rather than systematic population-based sampling, preclude conclusions regarding the geographic origin, transmission pathways, temporal dynamics, prevalence, or geographic expansion of these viruses.

Whole-genome comparisons also identified nucleotide and amino-acid differences among the Brazilian viruses. Because the viruses characterized in this study were sequenced after propagation in embryonated chicken eggs and direct whole-genome sequencing of the original clinical specimens was not performed, individual sequence differences cannot be unequivocally attributed to variation present in vivo. Consequently, these differences are reported descriptively and are not interpreted as evidence of adaptive evolution or independent evolutionary trajectories.

In 2024, 18 years after the last reported occurrence of virulent NDV in Brazilian poultry, an outbreak was reported in commercial broilers. According to the official report, the causative genotype VI virus shared 95.15% F-gene nucleotide identity with a PPMV-1 strain detected in a pigeon in Southern Brazil in 2014 [13,14]. However, the genome sequence of the 2024 outbreak virus is not currently available, preventing direct phylogenetic comparison with the viruses characterized here and limiting inference regarding their epidemiological relationship. Availability of the 2024 outbreak-virus sequence, together with additional historical and prospective sequence and surveillance data from South America, would enable a more comprehensive assessment of the relationships among pigeon-associated genotype VI viruses detected in the region.

## 5. Conclusions

This study expands the available genomic information on pigeon-associated NDV in Brazil and reveals substantial genetic heterogeneity and phylogenetic structure within sub-genotype VI.2.1.2. The two viruses characterized here clustered with pigeon-origin NDV previously detected in Brazil, and analysis of the broader VI.2.1.2 dataset supported further subdivision according to established NDV classification criteria. The detection of genetically distinct pigeon-associated NDV in geographically separated locations, together with the recent detection of genotype VI NDV in Brazilian poultry, highlights the value of continued genomic surveillance in pigeons and poultry to improve characterization of viral diversity and distribution.

## Figures and Tables

**Table 1:** Nucleotide distance analysis of the complete fusion (F) gene comparing sequences from the two newly identified sub-genotypes (VI.2.1.2.1 and VI.2.1.2.2) with other sub-genotypes, including VI.2.1.2. The distances of the newly assigned sub-genotypes are in bold font.

VI.1		VI.2.1	VI.2.1.2	**VI.2.1.2.1**	**VI.2.1.2.2**	VI.2.1.1.2.1	VI.2.1.1.2.2	VI.2.2.1	VI.2.2.2
VI.2.1	0.072								
VI.2.1.2	0.073	0.067							
**VI.2.1.2.1**	**0.077**	**0.080**	**0.061**						
**VI.2.1.2.2**	**0.093**	**0.097**	**0.080**	**0.073**					
VI.2.1.1.2.1	0.070	0.068	0.069	**0.078**	**0.093**				
VI.2.1.1.2.2	0.078	0.074	0.081	**0.090**	**0.100**	0.051			
VI.2.2.1	0.059	0.073	0.081	**0.086**	**0.101**	0.071	0.081		
VI.2.2.2	0.067	0.082	0.085	**0.092**	**0.108**	0.079	0.089	0.059	
VI.2.1.1.1	0.079	0.074	0.073	**0.078**	**0.095**	0.058	0.070	0.078	0.086

## Data Availability

Assembled genomes were deposited in GenBank under the accession numbers PQ212861 and PQ212862.

## Abbreviations

The following abbreviations are used in this manuscript:

F	Fusion protein
HN	Hemagglutinin-neuraminidase protein
L	Large protein
M	Matrix protein
NDV	Newcastle disease virus
NP	Nucleoprotein
nt	nucleotides
PPMV-1	Pigeon paramyxovirus 1
RNA	Ribonucleic acid
rRT-PCR	reverse transcriptase real-time polymerase chain reaction
WOAH	World Organisation for Animal Health

## Appendix A

**Table:** Pairwise nucleotide identities among Newcastle disease virus (NDV) isolates belonging to sub-genotypes VI.2.1.2, VI.2.1.2.1, and VI.2.1.2.2. The table shows the mean and range of genetic distances calculated from complete fusion (F) gene sequences.

		1	2	3	4	5	6	7	8	9	10	11	12	13	14	15	16	17
1	PQ212862_pigeon/Brazil/34/2022																	
2	PQ212861_picazuro_pigeon/Brazil/105/2021	97.41																
3	KX097024_1_pigeon_Brazil_N1065_14_2014	97.28	97.9															
4	MZ458602.1_pigeon_Brazil_SP_CCZ002_2019	99.21	98	98														
5	OM418380_pigeon/Brazil/MP003/2018	96.71	97.5	97	97.3													
6	AY734536_pigeon_Argentina_Capital_3_1997	93.34	94	95	94	94.1												
7	JQ039387_pigeon_Nigeria_VRD08_37BRpe_7_9_2008	91.72	92.3	93	92.4	93.1	93.8											
8	JQ039391_pigeon_Nigeria_VRD07_231_2007	91.56	92.2	93	92.3	92.9	93.7	98.8										
9	MH996920_quail_Nigeria_VRD08_385_N23_2008	91.65	92.3	93	92.3	93	93.8	98.8	98.8									
10	HG424627_pigeon_Nigeria_NIE13_092_2013	91.42	92.1	93	92.1	92.9	93.2	98.4	98.3	98.4								
11	MH996992_pigeon_Nigeria_Alaba_rago_VRD32_15_32_2015	91.19	92.1	93	91.9	92.7	93.4	98.9	98.8	98.9	98.5							
12	HG326602_pigeon_Nigeria_NIE07_062_2007	91.04	92	92	91.7	92.7	93.5	94.2	94.1	94.2	93.6	94						
13	HG326601_pigeon_Nigeria_NIE07_061_2007	91.05	92	92	91.7	92.7	93.5	94.2	94.1	94.2	93.6	94	94					
14	HG326603_pigeon_Nigeria_NIE07_063_2007	91.03	92	92	91.7	92.7	93.5	94.2	94.1	94.2	93.6	94	98.8	98.2				
15	HG326604_pigeon_Nigeria_NIE09_1898_2009	90.55	91.7	92	91.3	92.5	93.2	93.9	93.8	93.9	93.3	94	92.7	92.1	91.5			
16	JX518532_laughing_dove_Kenya_B2_Isiolo_2012	90.62	91.6	92	91.3	92.2	93.9	93.9	93.9	93.9	93.5	94	97.6	97.5	97.6	97.3		
17	MN422262.1_pigeon_Iran_AMMM160_2019	89.36	90.5	91	89.9	91.3	92.3	92.6	92.6	92.6	91.7	92	95.7	95.7	95.7	95.3	95.7	
